# 超高效液相色谱-四极杆-飞行时间质谱法快速筛查和确证渔药中86种非法添加化学品

**DOI:** 10.3724/SP.J.1123.2021.11023

**Published:** 2022-06-08

**Authors:** Qingqing KE, Shiyan LI, Dingnan WANG, Qin ZHOU, Fan ZHOU, Yijiang BEI, Xiaoming CHEN, Yang WANG

**Affiliations:** 浙江省水产技术推广总站, 浙江 杭州 310023; Zhejiang Fisheries Technical Extension Center, Hangzhou 310023, China

**Keywords:** 超高效液相色谱-四极杆-飞行时间质谱, 非法添加药物, 渔药, 快速筛查, ultra performance liquid chromatography-quadrupole-time-of-flight mass spectrometry (UPLC-Q-TOF/MS), illegally added chemicals, fishery drugs, rapid screening

## Abstract

建立了超高效液相色谱-四极杆-飞行时间质谱法快速筛查与确证渔药中86种非法添加禁限用药物的方法。渔药以80%(v/v)乙腈水溶液进行提取,通过稀释降低基质效应,采用ACQUITY PREMIER HSS T3色谱柱进行分离,以甲醇和0.1%甲酸水溶液作为流动相进行梯度洗脱,采用电喷雾双喷离子源(Dual AJS ESI)正离子模式分析检测。建立了86种药物的一级精确质量数据库和二级碎片质谱库。在全扫描采集模式下,以化合物的色谱保留时间、精确质量数、同位素分布和同位素丰度比定性;在Target MS/MS采集模式下,通过二级碎片离子的匹配进一步确证化合物,以准分子离子峰的峰面积定量,实现渔药样品中多目标药物的快速定性定量分析。86种药物在各自的线性范围内均呈现良好的线性关系,相关系数均大于0.99,中草药制剂和抗生素粉剂的定量限(LOQ)范围分别为1~15 mg/kg和5~75 mg/kg,添加回收率范围为76.8%~112.1%,相对标准偏差(RSD, *n*=3)小于11.7%。该方法快速、简便、准确、灵敏,适用于不同种类渔药中禁限用非法添加药物的高通量筛查。将该方法应用于浙江省渔用投入品质量安全监督抽检项目中,共筛查60个样品,其中8种中草药制剂筛查出说明书中未明确标明的药物成分,1种抗生素粉剂未检出有效成分。该研究为渔药的质量安全监控提供了有效的技术手段。

## Abstract (en)

With the rapid expansion of fisheries, one of the most significant limitations to the sustainable development of fisheries in China is the quality and safety of fishery products owing to the abuse of fishery drugs and the use of illegal and/or restricted chemicals in fishery drugs. A range of chemicals that are potential hazards to fishery drugs were selected for screening in this study. A comprehensive analytical method was developed, based on ultra performance liquid chromatography-quadrupole-time-of-flight mass spectrometry (UPLC-Q-TOF-MS), for the rapid screening of 86 types of illegally added chemicals in fishery drugs. The fishery drug samples were extracted with 80% (v/v) acetonitrile aqueous solution and diluted to reduce matrix effects. The 86 target compounds were separated on an ACQUITY PREMIER HSS T3 column (100 mm×2.1 mm, 1.8 μm), with methanol and 0.1% formic acid as mobile phases, via gradient elution. The extract was directly analyzed by UPLC-Q-TOF-MS using electrospray ionization in the positive mode. The external standard method was used for quantification. In this study, the extraction reagent and purification procedure were selected to develop a simple and effective pre-treatment protocol. The effects of the chromatographic column, mobile phase, and fragmentation voltage on the separation and sensitivity of the 86 substances were evaluated to determine the optimum instrument conditions. An accurate mass database and fragment ion library were created for the rapid qualitative and quantitative analysis of the 86 illegally added chemicals in fishery drugs. The retention time, isotopic abundance and spacing, and precise mass of the principal diagnostic ion for each analyte were used for identification. The information on the fragment ions obtained from the target MS/MS profiles was compared with that from a database to ensure the accuracy of the qualitative results. The chromatographic peak area of each target analyte was used for quantification. The analytical detection was based on the retention time deviation of ±0.35 min, accurate mass deviation of ±10×10^-6^, and major adduct forms, including [M+H]^+^, [M+Na]^+^, and [M+NH_4_]^+^. To evaluate the matrix effects of the 86 target chemicals at varied dilution ratios, two types of antibiotics and four types of Chinese herbal medicines were selected as typical samples. Considering the instrument tolerance as well as sensitivity and accuracy of the procedure, the recommended dilution ratios for antibiotics and Chinese herbal medicines were 50 times and 10 times, respectively. Two different types of calibration curves were prepared; one was the solvent calibration curve for antibiotics and the other was the matrix calibration curve for Chinese herbal medicines. For a given concentration, the calibration curves of the 86 target chemicals were linear with correlation coefficients of at least 0.99. The recoveries ranged from 76.8% to 112.1% with relative standard deviations (RSDs) (*n*=3) of less than 11.7%. The limit of quantification (LOQ) ranges of the compounds in Chinese herbal medicines and antibiotics were 1-15 mg/kg and 5-75 mg/kg, respectively. To evaluate the screening detection limits (SDLs) of each compound, a mixed standard solution was added to a fishery drug sample at varied concentrations. The SDL ranges of the compounds in Chinese herbal medicines and antibiotics were 1-15 and 5-50 mg/kg, respectively. This approach resulted in SDLs that satisfy the actual screening requirements. Because of its rapid nature, simplicity, accuracy, and sensitivity, the method may be used in the high-throughput screening and identification of illegally added chemicals in many types of fishery drugs. This method was applied to a monitoring project for the quality and safety of fishery inputs in Zhejiang Province. Sixty fishery drug samples were evaluated, among which eight Chinese herbal medicine samples were found to contain unspecified ingredients and one antibiotic sample was found to be free of any active substances. Thus, an effective technical method to monitor the quality and safety of fishery drugs was developed.

我国是水产品生产和出口大国,2019年水产养殖总产量达5079万吨,占渔业总产量的78.4%,出口总额达206.6亿美元,养殖量位居世界首位^[1]^。然而,伴随着渔业的快速发展,渔药滥用及渔药中禁限用药物非法添加引起的水产品质量安全问题已成为阻碍我国渔业健康可持续发展的最大制约因素之一。自2004年以来,国家和农业行政部门已经陆续发布了《无公害食品渔用药物使用准则》、250公告、2262公告、GB 31650-2019等多条法规标准对渔业生产中的禁限用药物做出了相应的规定。然而长期以来,由于市场无序竞争和管理制度上的缺陷等原因,渔药市场秩序混乱,产品质量参差不齐^[2⇓-4]^。具体表现为:以“非药品”的形式逃避监管;随意添加禁限用药物;中草药类产品中添加抗生素成分;为了降低成本,减少或者不添加有效成分等。这些成分不明、质量良莠不齐的渔药会产生治疗效果下降、水产品药物残留超标等问题,给养殖户带来极大的困扰,同时威胁消费者的身体健康,影响水产品的对外出口贸易。“问题渔药”已成为影响水产行业可持续发展的重要问题。2021年农业农村部发布1号文件《关于加强水产养殖用投入品监管的通知》,要求加大假、劣渔药处置,连续3年开展渔药等投入品相关违法行为专项整治行动。

目前我国渔药中多种禁限用药物非法添加的筛查尚无相关标准,相关技术研究较少^[5⇓⇓-8]^,主要采用液相色谱法和液相色谱-串联质谱法。这些方法存在检测的渔药品种单一且筛查药物覆盖面较窄,未考察基质效应对检测灵敏度和准确度的影响等问题。近年来,超高效液相色谱-四极杆-飞行时间质谱(UPLC-Q-TOF/MS)技术因具有高特异性、高质量分辨率、高通量、高扫描速度等优势开始在残留分析领域得到应用。国内外关于Q-TOF/MS多组分药物残留分析技术的研究多集中在环境、医药和食品安全等领域^[9⇓⇓⇓⇓-14]^,目前尚无采用Q-TOF/MS技术检测渔药中禁限用药物非法添加的文献报道。本研究结合水产养殖特点,有针对性地选取了渔药中可能存在风险隐患的多种禁限用药物作为筛查对象,通过稀释降低基质效应,基于UPLC-Q-TOF/MS技术,建立了渔药中86种非法添加禁限用药物的快速筛查和确证的方法。本方法具有适用范围广、前处理简单高效、分析通量高、灵敏准确等优点。本方法的建立为渔药的有效监管提供了技术支撑,对保障水产品质量安全具有重要意义。

## 1 实验部分

### 1.1 仪器、试剂与材料

Agilent 1290-6540液相色谱-四极杆-飞行时间质谱仪配电喷雾双喷离子源(Dual AJS ESI)(美国安捷伦公司); Sartorius 2202S型电子天平(德国赛多利斯有限公司);菲恰尔TDL-5A离心机(上海菲恰尔分析仪器有限公司); Centrifuge 5427R高速冷冻离心机(德国Eppendorf公司); Milli-Q超纯水系统(美国Millipore公司); 0.22 μm 聚四氟乙烯(PTFE)滤膜(美国PALL公司)。

86种化合物标准物质(包括19种磺胺类、16种喹诺酮类、18种头孢类、13种激素类、4种硝基呋喃类、3种四环素类、1种苯二氮卓类、6种大环内酯类、4种硝基咪唑类、2种三苯甲烷类等各类别的混合标准溶液,质量浓度均为100 mg/L)(天津阿尔塔科技有限公司),具体信息见表1。

**表1:** 86种化合物的分子式、保留时间、质量偏差、检索得分和其他质谱参数

Compound	Formula			*t*_R_/ min		Precursor ion (*m/z*)		Fragment ions (*m/z*)		Mass error/ 10^-6^		Score
Sulfonamides												
Sulfadiazine (磺胺嘧啶)	C_10_H_10_N_4_O_2_S			4.546		251.05949		156.01120, 108.04430, 92.04940		-1.28		97.26
Sulfathiazole (磺胺噻唑)	C_9_H_9_N_3_O_2_S_2_			5.091		256.02121		156.01144, 108.04450, 92.04980		-0.73		97.21
Sulfapyridine (磺胺吡啶)	C_11_H_11_N_3_O_2_S			5.369		250.06441		184.08681, 92.04960, 65.03890		-0.91		97.12
Sulfamerazine (磺胺甲基嘧啶)	C_11_H_12_N_4_O_2_S			5.814		265.07587		156.01125, 108.04459, 65.03888		-0.92		97.31
Sulfamethazine (磺胺二甲基嘧啶)	C_12_H_14_N_4_O_2_S			6.792		279.09201		186.03365, 124.08719, 92.04982		-0.56		98.76
Sulfamonomethoxine (磺胺间甲氧嘧啶)	C_11_H_12_N_4_O_3_S			7.337		281.07111		156.01157, 126.06642, 92.04983		-0.65		97.35
Sulfamethizole (磺胺甲噻二唑)	C_9_H_10_N_4_O_2_S_2_			6.636		271.03183		156.01128, 108.04436, 92.04963		-0.90		95.09
Sulfameter (磺胺对甲氧嘧啶)	C_11_H_12_N_4_O_3_S			6.558		281.07098		156.01170, 126.06651, 92.04993		-1.10		97.08
Sulfachloropyridazine (磺胺氯哒嗪)	C_10_H_9_ClN_4_O_2_S			7.170		285.02069		156.01106, 92.04995, 65.03902		-0.93		98.85
Sulfamethoxypyridazine (磺胺甲氧哒嗪)	C_11_H_12_N_4_O_3_S			6.936		281.07011		92.04993, 156.01170		-0.93		98.35
Sulfadoxine (磺胺邻二甲氧嘧啶)	C_12_H_14_N_4_O_4_S			7.603		311.08188		156.01164, 92.04980, 65.03902		-1.37		98.10
Sulfadimethoxine (磺胺间二甲氧嘧啶)	C_12_H_14_N_4_O_4_S			8.426		311.08188		156.07678, 92.04969, 65.03891		-1.03		96.98
Sulfamethoxazole (磺胺甲基异噁唑)	C_10_H_11_N_3_O_3_S			7.259		254.05981		156.01135, 108.04437, 92.04968		-1.07		98.41
Sulfisoxazole (磺胺二甲异噁唑)	C_11_H_13_N_3_O_3_S			7.592		268.07556		156.01135, 113.07117, 92.04985		-1.15		99.40
Sulfabenzamide (苯甲酰磺胺)	C_13_H_12_N_2_O_3_S			7.859		277.06448		156.01143, 92.04991, 65.03888		-1.35		98.38
Sulfaquinoxaline (磺胺喹恶啉)	C_14_H_12_N_4_O_2_S			8.615		301.07587		108.04438, 92.04956, 65.03878		-1.02		97.12
Sulphacetamide (磺胺醋纤)	C_8_H_10_N_2_O_3_S			3.535		215.04842		156.01102, 108.04445, 92.04966		-0.92		98.69
Trimethoprim (甲氧苄氨嘧啶)	C_14_H_18_N_4_O_3_			6.470		291.14603		123.06687, 81.04508, 68.03694		-0.49		81.21
Sulfaphenazole (磺胺苯吡唑)	C_15_H_14_N_4_O_2_S			8.182		315.09167		160.08691, 92.04948, 65.03879		-0.79		98.67
Quinolones												
Enrofloxacin (恩诺沙星)	C_19_H_22_FN_3_O_3_			7.259		360.16991		342.16180, 286.09937, 84.08111		-0.50		99.71
Norfloxacin (诺氟沙星)	C_16_H_18_FN_3_O_3_			7.025		320.14069		303.12708, 234.10332, 70.06547		-1.30		97.22
Pefloxacin (培氟沙星)	C_17_H_20_FN_3_O_3_·CH_4_SO_3_			6.914		334.14886		316.14542, 232.05943, 70.06556		-0.79		99.38
Ciprofloxacin (环丙沙星)	C_17_H_18_FN_3_O_3_			7.159		332.14063		316.14615, 231.05635		-0.49		90.10
Ofloxacin (氧氟沙星)	C_18_H_20_FN_3_O_4_			6.892		362.15140		344.14087, 261.10367, 58.06546		-0.99		98.35
Sarafloxacin (沙拉沙星)	C_20_H_17_F_2_N_3_O_3_			7.570		386.13168		368.12033, 299.09836, 270.09607		-0.5		99.42
Enoxacin (依诺沙星)	C_15_H_17_FN_4_O_3_			6.936		321.13754		303.12589, 232.05235, 204.05697		-3.92		96.25
Lomefloxacin (洛美沙星)	C_17_H_19_F_2_N_3_O_3_			7.370		352.14673		334.13544, 308.15662, 265.11450		-1.11		98.25
Nalidixic acid (萘啶酸)	C_12_H_12_N_2_O_3_			9.538		233.09242		215.08206, 187.05052, 131.06062		-0.95		83.08
Oxolinic acid (恶喹酸)	C_13_H_11_NO_5_			8.771		262.07141		244.06110, 216.02902, 160.03928		-1.11		84.53
Flumequin (氟甲喹)	C_14_H_12_FNO_3_			9.682		262.08786		244.07687, 202.03030, 174.03494		-0.78		88.35
Danofloxacin (达氟沙星)	C_19_H_20_FN_3_O_3_			7.292		358.15692		340.14630, 255.05676, 82.06566		-0.65		88.45
Difloxacin (双氟沙星)	C_21_H_19_F_2_N_3_O_3_·HCl			7.448		400.14807		382.13608, 299.09827, 58.06530		-0.8		99.55
Orbifloxacin (奥比沙星)	C_19_H_20_F_3_N_3_O_3_			7.459		396.15268		352.16428, 295.10602, 267.03815		-1.00		94.54
Sparfloxacin (司帕沙星)	C_19_H_22_F_2_N_4_O_3_			7.993		393.17398		349.18417, 292.12643, 58.06548		-0.84		96.84
Fleroxacin (氟罗沙星)	C_17_H_18_F_3_N_3_O_3_			6.659		370.13788		326.14777, 269.09048, 58.06532		-0.93		96.62
Cephalosporins												
Cephalexin (头孢氨苄)	C_16_H_17_N_3_O_4_S			6.992		348.10022		140.01656, 106.06514, 68.05019		-1.06		89.72
Cephapirin (头孢匹林钠)	C_17_H_16_N_3_O_6_S_2_Na			5.585		424.06357		292.05737, 181.04324, 152.01637		-0.82		97.57
Cefaclor (头孢克洛)	C_15_H_14_ClN_3_O_4_S			6.603		368.04694		174.54530, 106.06565		-0.07		96.51
Cefixime (头孢克肟)	C_16_H_15_N_5_O_7_S2			7.337		454.04984		285.02887, 210.02115, 126.01183		-0.97		97.63
Cephradine (头孢拉定)	C_16_H_19_N_3_O_4_S			7.381		350.18863		191.08199, 108.08082, 91.05441		-0.32		84.89
Cefquinome (头孢喹肟)	C_22_H_22_N_6_O_5_S_2_			6.347		529.13214		134.09663, 167.02542		0.31		99.49
Cefetamet pivoxyl (头孢他美酯)	C_20_H_25_N_5_O_7_S_2_			9.983		512.12869		398.05936, 241.03958, 57.07028		-0.36		99.58
Cefazolin (头孢唑啉)	C_14_H_14_N_8_O_4_S_3_			7.415		455.03787		323.05524, 295.06052, 156.01146		-0.63		92.67
Desacetylcefotaxime (3-去乙酰基头孢噻肟)	C_14_H_15_N_5_O_6_S_2_			5.469		414.0545		156.02243, 126.01189, 60.04457		-0.42		99.60
Cefamandole lithium (头孢孟多锂)	C_18_H_17_N_6_O_5_S_2_Li			8.059		463.08127		347.06961, 158.02698, 68.04770		0.78		94.31
Compound	Formula			*t*_R_/min		Precursor ion (*m/z*)		Fragment ions (*m/z*)		Mass error/ 10^-6^		Score
Cefminox sodium (头孢米诺钠盐)	C_16_H_20_N_7_NaO_7_S_3_			4.635		520.07169		161.03818, 215.04808		0.68		97.37
Cefoperazone sodium (头孢哌酮钠)	C_25_H_26_N_9_NaO_8_S_2_			7.781		646.14758		530.13062, 290.11304, 143.08093		1.20		92.83
Cefadroxil (头孢羟氨苄)	C_16_H_17_N_3_O_5_S			4.680		364.09638		208.02491, 114.00074, 68.04988		-0.63		84.34
Ceftiofur (头孢噻呋)	C_19_H_17_N_5_O_7_S_3_			8.815		524.03809		241.03912, 210.02058, 126.01196		0.28		97.01
Cefotaxime (头孢噻肟)	C_16_H_17_N_5_O_7_S_2_			7.203		456.06531		323.05579, 295.05969, 156.01146		-0.56		98.26
Ceftazidime (头孢他啶)	C_22_H_22_N_6_O_7_S_2_			5.747		547.10073		396.07877, 277.02289, 167.02718		0.87		97.25
Cephalonium (头孢洛宁)	C_20_H_18_N_4_O_5_S_2_			6.114		459.07953		337.03067, 152.01624, 123.05494		0.12		99.55
Cefpirome (头孢喹肟)	C_22_H_22_N_6_O_5_S_2_			5.558		515.11633		396.04276, 324.05823, 120.08104		0.56		98.60
Hormones												
Medroxyprogesterone 17-acetate	C_24_H_34_O_4_			11.317		387.25415		285.22110, 123.08038, 97.06510		-1.18		98.99
(醋酸甲羟孕酮)												
17-Methyltestosterone (甲基睾酮)	C_20_H_30_O_2_			11.205		303.23282		227.17902, 109.06502, 97.06518		-1.10		98.12
Testosteronepropionate (丙酸睾丸素)	C_22_H_32_O_3_			11.572		345.23514		97.06500, 109.06480		-1.14		98.62
Norgestimate (炔诺肟酯)	C_23_H_31_NO_3_			11.417		370.2388		124.07552, 98.06010, 79.05455		-1.51		98.28
Norethindrone (炔诺酮)	C_20_H_26_O_2_			10.872		299.20129		231.17410, 109.06481, 83.04930		-0.65		97.44
Testosterone (睾酮)	C_19_H_28_O_2_			11.072		289.21695		109.06510, 97.06523		-1.00		98.57
Nortestosterone (诺龙)	C_18_H_26_O_2_			10.883		275.20148		257.18985, 109.06498, 83.04929		-0.96		98.29
Trenbolone (群勃龙)	C_18_H_22_O_2_			10.650		271.1701		199.11153, 107.04932, 83.04940		-4.29		97.20
D-(-)-Norgestrel (甲基炔酮)	C_21_H_28_O_2_			11.150		313.21692		245.19000, 109.06498, 83.04930		-1.27		98.66
Megestrol (甲地孕酮)	C_22_H_30_O_3_			11.228		343.22778		267.17401, 187.11162, 97.06499		-1.27		98.20
Hydrocortisone (氢化可的松)	C_21_H_30_O_5_			10.038		363.21699		327.19510, 121.06483, 97.06495		-1.09		98.59
Cortisone (可的松)	C_21_H_28_O_5_			9.821		361.20181		163.11154, 121.06488, 93.07002		-1.13		98.94
Prednisone (泼尼松)	C_21_H_26_O_5_			9.727		359.18607		265.15845, 171.08022, 147.08022		-0.96		98.79
Nitrofurans												
Nitrofurazone (呋喃西林)	C_6_H_6_N_4_O_4_			6.465		199.04688		182.01959, 108.03228, 54.01043		-1.33		99.73
Furazolidone (呋喃唑酮)	C_8_H_7_N_3_O_5_			6.558		226.04655		139.01385, 122.01112, 67.04184		-0.92		99.57
Nitrofurantoin (呋喃妥因)	C_8_H_6_N_4_O_5_			6.476		239.04124		103.07568, 59.04915		0.50		99.43
Furaltadone (呋喃它酮)	C_13_H_16_N_4_O_6_			4.153		325.11456		252.09782, 128.10684, 100.07582		-0.93		93.61
Triphenylmethane												
Leucomalachite green (孔雀石绿)	C_23_H_26_N_2_			11.222		331.21772		313.16995, 208.11189		-0.92		99.17
Malachite green (无色孔雀石绿)	C_23_H_24_N_2_			9.977		329.20185		313.16995, 208.11189		-0.52		83.43
Tetracyclines												
Oxytetracycline (四环素)	C_22_H_24_N_2_O_8_			7.081		461.14818		201.05482, 426.11868		0.49		98.75
Tetracycline (土霉素)	C_22_H_24_N_2_O_9_			6.970		445.15327		154.04965, 410.12381		0.33		91.11
Chlortetracycline (金霉素)	C_22_H_23_ClN_2_O_8_			8.059		479.11429		154.04990, 444.08395		0.52		95.27
Macrolides												
Erythromycin (红霉素)	C_37_H_67_NO_13_			9.749		734.46907		576.37170, 158.1160		-0.19		86.42
Roxithromycin (罗红霉素)	C_41_H_76_N_2_O_15_			10.338		837. 5318		679.43780, 494.3355		-0.41		87.31
Lincomycin (林可霉素)	C_18_H_34_N_2_O_6_S			6.281		407.2137		359.21640, 126.1274		-0.95		86.90
Clarithromycin (克拉霉素)	C_38_H_69_NO_13_			10.272		748.47689		407.22150, 126.1748		-0.66		89.30
Oleandomycin (磷酸竹桃霉素)	C_35_H_64_NO_16_P			9.249		688.41938		544.31530, 158.1753		-0.63		84.78
Clindamycin (克林霉素)	C_18_H_33_ClN_2_O_5_S			8.930		425.17987		350.22220, 174.1116		-0.89		80.87
Nitroimidazoles												
Dimetriidazole (地美硝唑)	C_5_H_7_N_3_O_2_			4.414		142.05383		96.01963, 81.15461		-0.46		99.51
Dimetridazole-2-hydroxy (羟甲基甲硝咪唑)	C_5_H_7_N_3_O_3_			3.599		158.04874		112.06292, 140.04510		-0.32		99.81
Hydroxymetronidazole (羟基甲硝唑)	C_6_H_9_N_3_O_4_			2.995		188.05931		123.05501, 126.02953		-1.27		99.66
Metronidazole (甲硝唑)	C_6_H_9_N_3_O_3_			4.054		172.06439		111.04098, 128.04526		-0.85		98.65
Benzodiazepines												
Diazepam (地西泮)	C_16_H_13_ClN_2_O			10.855		285.07164		154.04146, 193.08847		-0.80		99.06

乙腈和甲醇(色谱纯,美国TEDIA公司);甲酸(色谱纯,美国ROE公司);吸附剂*N*-丙基乙二胺(PSA)、十八烷基键合硅胶(C_18_)、石墨化炭黑(GCB)(美国Agilent公司)。

### 1.2 标准溶液的配制

准确量取18种头孢类标准物质,以水-乙腈混合溶液(3∶1, v/v)为溶剂,配制成50 mg/L的混合标准储备溶液,小体积分装于棕色试剂瓶后-80 ℃保存。准确移取18种头孢类混合标准储备液,水-乙腈混合溶液(3∶1, v/v)为溶剂,配制成1 mg/L的18种头孢类标准中间液,现配现用。分别准确量取适量除头孢外的各类混合标准物质(即磺胺类、喹诺酮类、激素类、硝基呋喃类、四环素类、硝基咪唑类、大环内酯类、三苯甲烷类混合标准溶液及苯二氮卓类标准溶液),以甲醇为溶剂,配制9组10 mg/L的混合标准储备液,置于棕色试剂瓶中-20 ℃保存。分别准确移取适量除头孢外各类混合标准储备液,以甲醇为溶剂,配制成68种1 mg/L的混合标准中间液,置棕色试剂瓶中于-20 ℃保存。分别移取一定体积的18类头孢类化合物和68种化合物的混合标准中间液,用10%(v/v)乙腈水溶液进行稀释,配制成100 μg/L的86种化合物的混合标准溶液。

### 1.3 样品制备

所有渔药样品采自浙江省内各水产养殖场和渔药商店,包括抗生素粉剂和中草药制剂等,所有样品常温密封保存,待检测。

### 1.4 样品前处理

#### 1.4.1 样品提取

准确称取粉末状渔药100 mg(液态药物用移液枪准确移取100 μL)于50 mL离心管中,加入2 mL超纯水,涡旋2 min,加入8 mL乙腈,40 ℃超声提取15 min, 12000 r/min离心5 min,待稀释。

#### 1.4.2 稀释净化

抗生素粉剂类渔药,准确移取提取液20 μL,用10%(v/v,下同)乙腈水溶液定容至1 mL,配制成稀释倍数为50倍的初始稀释液;中草药制剂准确移取提取液100 μL,用10%乙腈水溶液定容至1 mL,配制成稀释倍数为10倍的初始稀释液;上述稀释液均过0.22 μm微孔滤膜,上机测试。其中抗生素粉剂上机液进样时,每个样品间隔1针空白溶剂进样。

### 1.5 UPLC-Q-TOF/MS条件

#### 1.5.1 色谱条件

色谱柱为Waters ACQUITY PREMIER HSS T3(100 mm×2.1 mm, 1.8 μm);流动相A为0.1%甲酸水溶液,流动相B为甲醇;流速为0.4 mL/min;柱温为40 ℃;进样量为2 μL。梯度洗脱程序设定:0~1 min, 3%B; 1~5 min, 3%B~20%B; 5~10 min, 20%B~75%B; 10~10.5 min, 75%B~100%B; 10.5~14 min, 100%B; 14~14.5 min, 100%B~3%B; 14.5~15 min, 3%B。

#### 1.5.2 质谱条件

离子源:Dual AJS ESI源;扫描方式:正离子全扫描;毛细管电压:4000 V;鞘气温度:350 ℃;鞘气流速:12.0 L/min;干燥气流速:8.0 L/min;干燥气温度:325 ℃;诱导解离电压:150 V。一级质谱数据采集为单极质谱全扫描模式,全扫描范围*m/z* 50~1000,采集速率为2 spectra/s。二级质谱数据采集为目标离子采集模式,扫描范围*m/z* 50~1000,采集速率为2 spectra/s,碰撞能量为10、20和40 eV。采集数据时采用参比液(嘌呤,C_5_H_4_N_4_,其离子精确相对质量为121.050863)实时质量校准。

#### 1.5.3 筛查参数

采用Agilent Mass Hunter Workstation Software(Version B 5.00)软件完成各化合物数据的采集与处理,Agilent Mass Hunter PCDL Manager(B 4. 00)软件完成化合物筛查数据库的建立。匹配总得分涉及质量得分、同位素强度得分、同位素间距得分、保留时间等因素,四者的权重值均100。筛查列表以±0.02 Da质量宽度提取化合物的分子离子峰。数据库筛查以±10×10^-6^(±10 ppm)精确质量偏差提取化合物的分子离子峰,保留时间限定误差±0.35 min,离子化形式选择+H、+NH_4_、+Na模式。

## 2 结果与讨论

### 2.1 色谱-质谱条件的优化

#### 2.1.1 色谱柱的选择

本研究考察了ACQUITY PREMIER HSS T3(100 mm×2.1 mm, 1.8 μm,简称T3柱)、ZORBAX Eclipse Plus C_18_(100 mm×3.0 mm, 1.8 μm)和ZORBAX Eclipse Plus C_18_ Narrow Bore RR (100 mm×2.1 mm, 3.5 μm) 3种色谱柱对86种化合物的分离情况和检测灵敏度。结果表明,大部分化合物在T3柱上的灵敏度更高,尤其是头孢类药物和四环素类药物,其在T3柱上的响应是其他两根色谱柱上的2~4倍。相较于其他色谱柱,T3柱对于部分极性较大的药物具有更强的保留能力,同时新型PREMIER T3柱在柱管内侧和填料之间采用了MAX PEAK HPS表面涂层技术,可通过减少四环素类等化合物与色谱柱表面金属离子的相互作用,改善化合物的峰形,提高检测的灵敏度。

#### 2.1.2 流动相的选择

选择PREMIER T3色谱柱,比较了甲醇-水体系和乙腈-水体系在梯度洗脱条件下对86种化合物分离效果和灵敏度的影响。结果表明,在ESI^+^模式下,流动相中加入0.1%的甲酸可提高目标药物的离子化效率,明显改善酸碱化合物的峰形。乙腈-水体系无法将3种磺胺同分异构体(C_11_H_12_N_4_O_3_S)中的磺胺对甲氧嘧啶和磺胺甲氧哒嗪分开,而甲醇-水体系则可以很好地将这两种化合物分开。甲醇极性强,作为流动相对两性化合物和极性大的化合物更有优势,同时洗脱能力较弱,能够对化合物较好保留。此外,在本实验条件下,甲醇还可以显著提高头孢类(平均响应提高3倍)、四环素类(平均响应提高3.5倍)和磺胺类(平均响应提高2倍)等化合物的灵敏度。因此本方法选择甲醇-0. 1%甲酸水溶液作为流动相。图1为3种磺胺类药物的同分异构体(C_11_H_12_N_4_O_3_S)分别在乙腈-0. 1%甲酸水溶液体系和甲醇-0. 1%甲酸水溶液体系中的提取离子色谱图。图2为86种化合物混合标准溶液的提取离子色谱图,在1.5节条件下,14 min内可完成86种化合物的分析,且目标物的峰形较好,保持了较高的检测灵敏度。

**图1:**
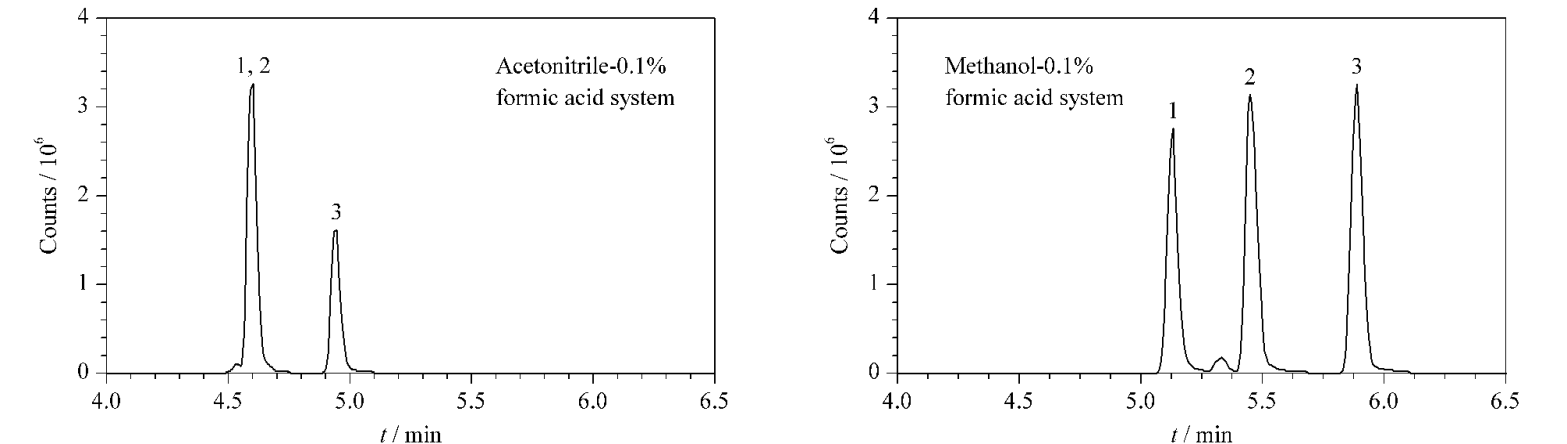
不同流动相体系下3种磺胺类药物同分异构体的提取离子色谱图

**图2:**
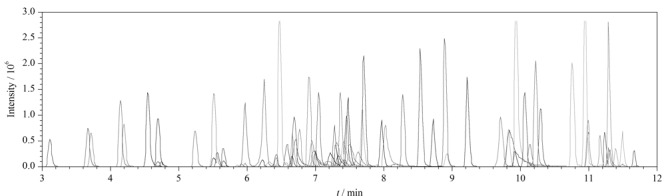
86种化合物的提取离子流色谱图

#### 2.1.3 质谱参数优化

飞行时间质谱仪毛细管出口区域的碰撞诱导解离电压(Fragmentor)能够影响离子的传输效率,Fragmentor过低时不利于离子聚焦和传输,Fragmentor过高时会造成离子的源内裂解,两种情况都会对检测的灵敏度产生影响^[15]^。本研究分别优化了10类86种化合物的诱导解离电压在70~210 V时的分子离子峰响应情况。图3为每类化合物在不同强度诱导解离电压下的分子离子峰的平均响应强度。结果显示,诱导解离电压为150 V时,10类86种化合物均能获得较高强度的响应值,因此本实验选择诱导解离电压为150 V。

**图3:**
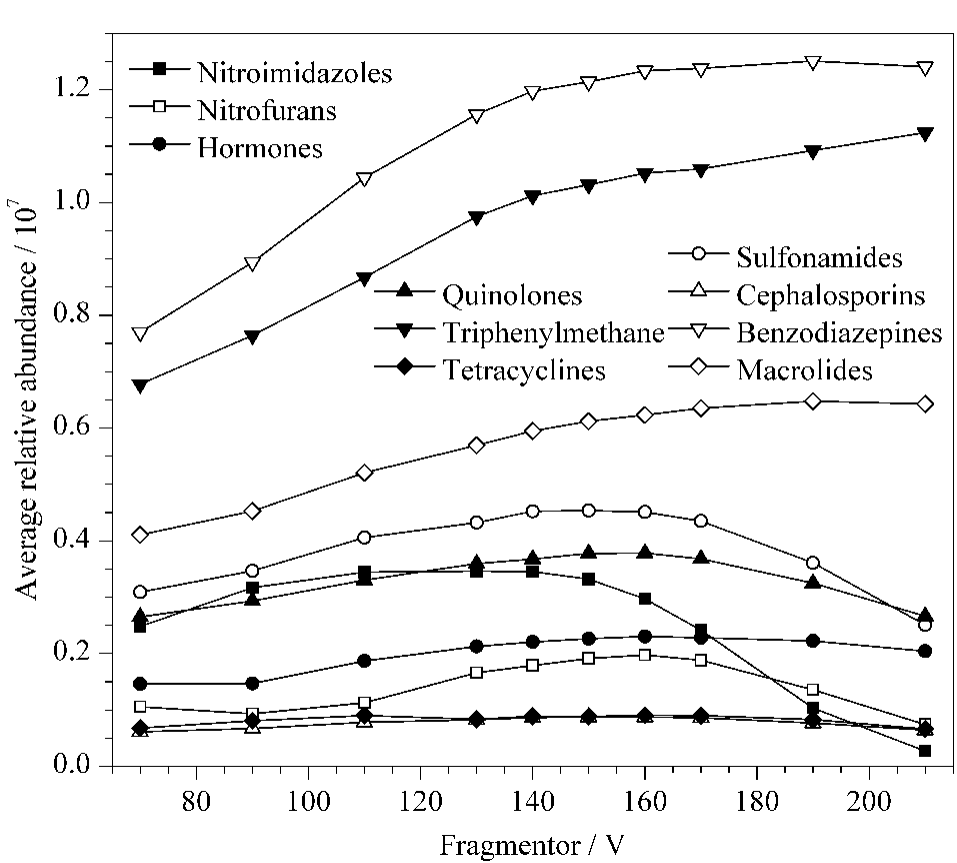
碎裂电压为70~210 V时10类化合物的平均响应强度趋势

### 2.2 筛查信息数据库建立

#### 2.2.1 一级精确质量数据库的建立

化合物筛查数据库是筛查目标化合物的判定依据,在最佳的色谱-质谱条件下,对86种化合物进行Q-TOF/MS一级质谱全扫描分析获取信息,分别将86种化合物的色谱峰对应的化合物名称、分子式、母离子精确质量数及保留时间等信息导入PCDL数据库软件,得到化合物的一级精确质量数据库,用于软件初步筛查分析。

#### 2.2.2 二级碎片离子质谱图数据库的建立

在1.5节条件下,对已获得精确质量数的母离子作不同碰撞能量(10、20、40 eV)的Targeted MS/MS二级质谱扫描,采集并分析所有二级碎片离子质谱图,得到86种化合物离子的质谱图数据库,用于精确质量数据库初筛结果的最终确认。另外,将相对丰度在10%以上的碎片离子作为该化合物的定性点,取定性点最多的碰撞能量作为其确证的最佳条件。86种化合物的定性点均达到4个以上,满足欧盟2002/657/EC决议对质谱分析方法的规定^[16]^。UPLC-Q-TOF/MS获得的定性位点远远高于LC-MS/MS的4个位点,从而大大降低了出现假阳性结果的概率,提高了定性结果的可信度。表1列出了86种化合物的精确质量数、保留时间、母离子和相对丰度最高的2~3个特征离子碎片、质量偏差和一级库检索得分等信息。

### 2.3 样品前处理条件的选择和优化

#### 2.3.1 提取试剂的选择

本研究涉及激素类、磺胺类、氟喹诺酮类、硝基咪唑类、大环内酯类、头孢类、四环素类等多类化合物,化合物间化学性质差异较大,应选择合适的提取溶剂以满足所有化合物的提取需求。实验分别比较了甲醇、乙腈、80%乙腈水溶液作为提取溶剂时的提取效果。结果如图4所示,纯甲醇或纯乙腈对头孢类、喹诺酮类、四环素类药物提取效果较差,80%乙腈水溶液提取效果最佳。在80%乙腈水溶液中分别加入0.2%、0.1%、0.05%的甲酸进行考察,实验发现,甲酸的加入会增强部分头孢类、四环素类、孔雀石绿和喹诺酮类药物的响应,但磺胺类和红霉素类药物响应有较大幅度降低,尤其是红霉素。红霉素在酸性条件下不稳定、易分解^[17]^。提取体系存在0.05%甲酸时红霉素的响应比无甲酸时的响应降低70%。综上,选择80%乙腈水溶液作为提取试剂。

**图4:**
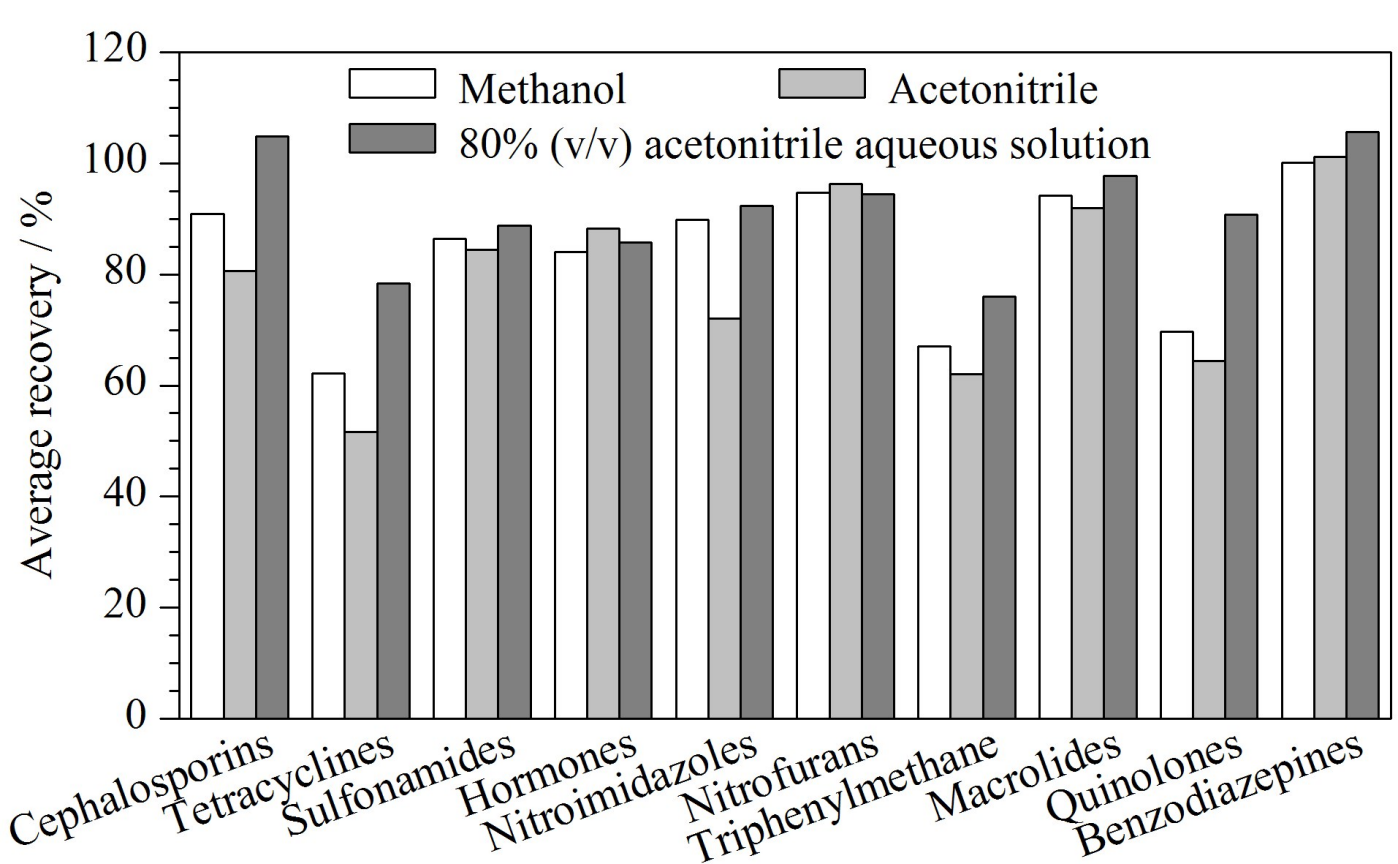
10类化合物在不同提取体系时的平均回收率

#### 2.3.2 净化方式的选择

研究比较了C_18_、PSA、GCB等3种吸附剂对渔药提取液的净化效果和对目标化合物回收率的影响。结果显示,GCB能有效地去除渔药中的色素,但对喹诺酮类药物具有极强的吸附。加入20 mg的GCB时,16种喹诺酮类化合物中有14种回收率低于10%。PSA能够去除极性干扰物质,但对四环素类、喹诺酮类和头孢类等化合物有较强的吸附。当加入50 mg的PSA时,14种头孢药物和3种四环素类药物完全被吸附,9种喹诺酮类药物回收率低于60%,此结果与郭海霞等^[18]^的实验结果一致。C_18_能够吸附脂肪等强疏水性干扰物,而渔药中草药中的主要干扰物质一般为色素和有机酸。虽然C_18_对86种化合物均无明显吸附,但通过净化前后化合物回收率的比对发现,C_18_对渔药基质无法起到较好的净化效果。渔药一般通过池塘泼洒或拌饲进行给药,若进行非法添加通常添加药物含量较高,本研究考虑采用直接稀释法以降低基质效应(ME)。

#### 2.3.3 稀释倍数和基质效应评价

渔药样品经过稀释后可以减少基质效应对检测结果的干扰,但稀释倍数过高会降低分析方法的灵敏度,因此需要对渔药在不同稀释倍数下的基质效应进行考察,以确定适宜的稀释倍数。本实验中基质效应通过如下公式计算:基质效应=(基质空白溶液加标信号强度/空白溶剂加标信号强度)×100%,当计算值为80%~120%时,认为基质效应不明显;小于80%时,表明存在明显的基质抑制作用;大于120%时,表明存在明显的基质增强作用^[19]^。抗生素粉剂和中草药制剂基质差异性较大,本研究同时对这两大类渔药进行了基质效应考察。抗生素粉剂选择了2种常见治疗渔药为代表样品(主要成分分别为恩诺沙星和红霉素),中草药制剂选取了4种渔用中草药为代表样品(主要成分基本涵盖渔药中草药制剂的常见药草成分)。按照1.4.1节处理获取提取液,以10%乙腈水溶液作为稀释液,考察了上述渔药提取液在2、5、10、20、50、100和200倍稀释倍数下86种化合物的基质效应。

结果如图5所示,2种抗生素粉剂基质干扰较小,在样品稀释倍数为5倍时,86种化合物即无明显的基质效应,但此时抗生素粉剂原药上机浓度过高,会对仪器造成污染。经验证,当抗生素粉剂稀释倍数为50倍时,在2 μL进样量情况下,通过上机时样品之间间隔1针空白试剂进样,即可消除色谱体系中原药残留对下个样品分析的影响。考虑到稀释倍数过高,会降低筛查灵敏度,实验中将抗生素粉剂的初始稀释倍数定为50倍,并通过上机时样品之间间隔空白试剂进样的方式消除针间污染,此时86种化合物均无明显的基质效应,直接采用试剂配制标准曲线外标法进行定量分析。4种中草药制剂基质较为复杂,在稀释倍数较低时(如2倍和5倍),基质效应显著的化合物数量较多,如3号中草药制剂在稀释5倍时,仅有51种化合物的基质效应在80%~120%范围内,当稀释倍数增加至10倍时,仅有少量化合物存在明显的基质效应。在本实验中为获得较低的筛查限(SDL),将中草药制剂类渔药的初始稀释倍数定为10倍,并通过基质匹配标准曲线法进行定量分析,以保证检测结果的准确性。

**图5:**
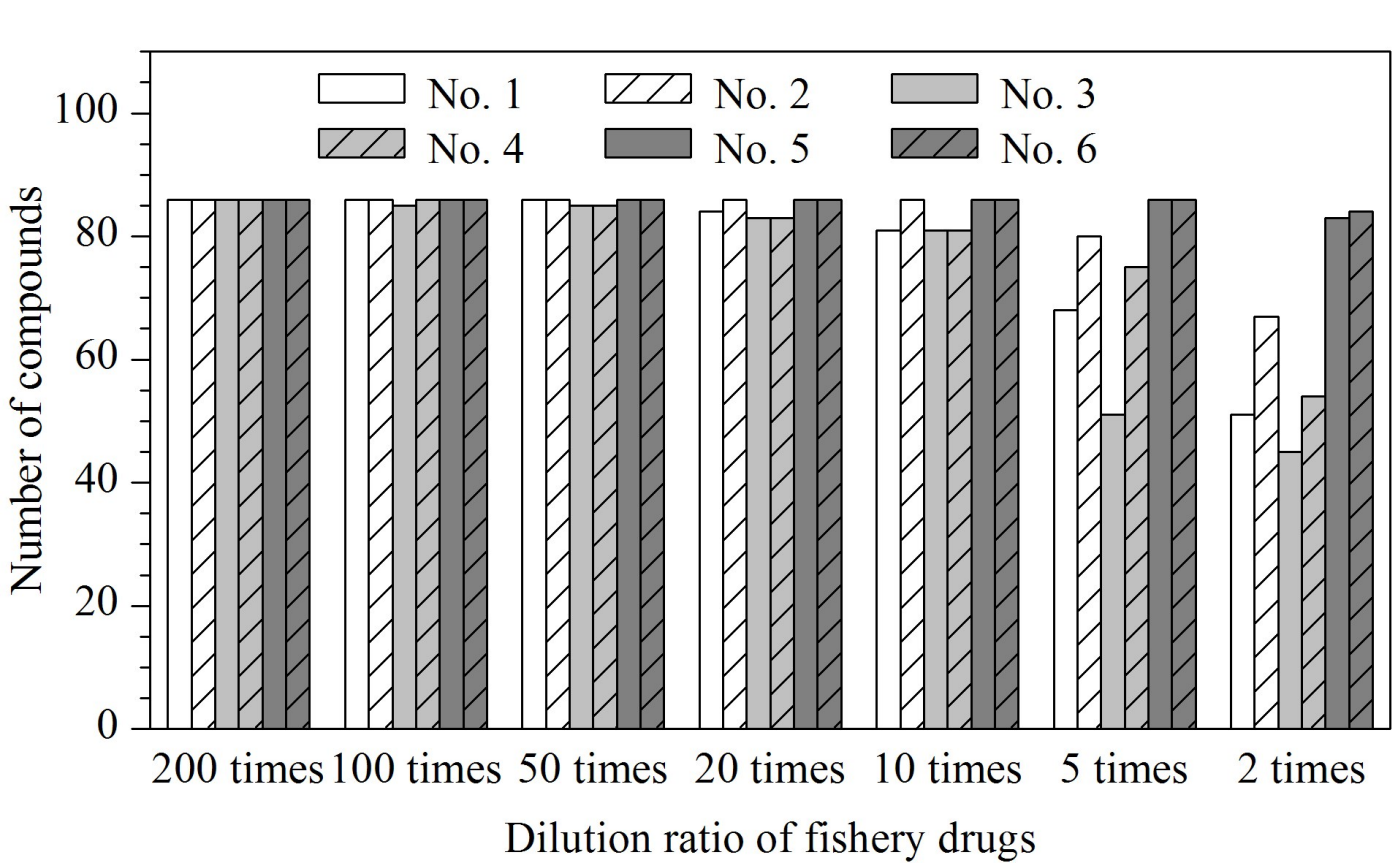
6种渔药在不同稀释倍数下基质效应在80%~120%之间的化合物数量

### 2.4 定性筛查与确证

实际样品经过前处理后首先通过一级质谱全扫描测定,获得的数据通过已建立的一级精确质量数据库在设定的检索参数下进行自动检索,软件根据实测离子与数据库中的精确质量偏差、保留时间、同位素分布和同位素比例4个因素的匹配程度进行打分,经优化,将检索得分≥80分的化合物,确定为疑似阳性。再利用二级质谱的碎片离子对疑似阳性样品进一步确证。样品经二次进样测定后,将样品的二级碎片离子信息与已建立的二级碎片离子质谱图数据库中碎片离子信息进行匹配,若有两个及两个以上的主要碎片离子匹配,则可确证为非法添加的药物。

以实际样品中检测到的磺胺类药物甲氧苄氨嘧啶为例,对实际样品前处理后进行MS测定,获得结果通过一级质谱库进行检索,检索得分为97分,保留时间和同位素比例及其分布与数据库中的理论值匹配良好,质量偏差为0.78×10^-6^,从而认为甲氧苄氨嘧啶为此渔药中疑似添加的药物。图6a为甲氧苄氨嘧啶的一级提取离子流色谱图,图6b为一级质谱同位素分布图。将样品的二级碎片离子信息与二级库中碎片离子信息进行匹配。图6c为实际样品中甲氧苄氨嘧啶和数据库中甲氧苄氨嘧啶的二级质谱对比结果,可以明显看出其主要的特征碎片与谱图库均匹配良好,二级得分为90分,故确定样品中含有甲氧苄氨嘧啶。

**图6:**
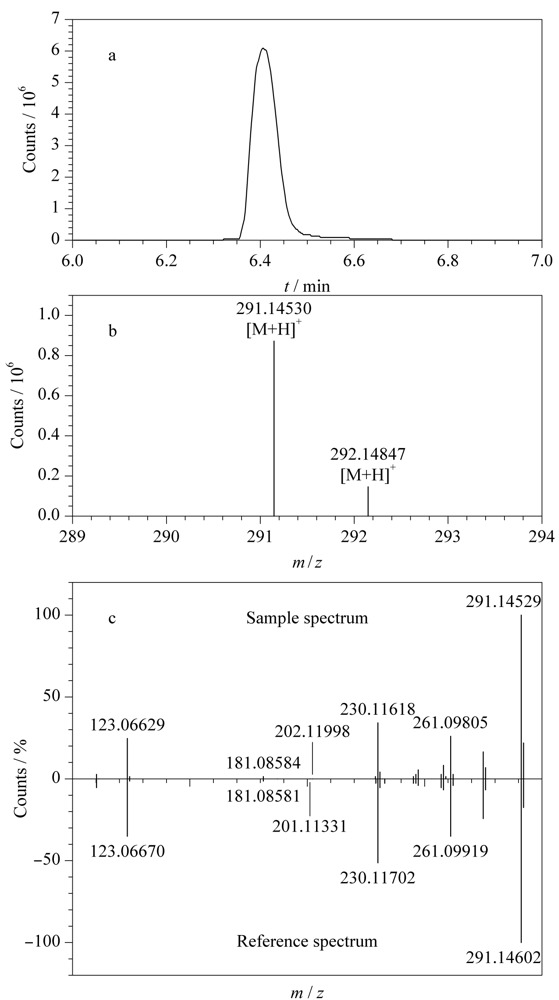
实际渔药样品中甲氧苄氨嘧啶的(a)提取离子色谱图、 (b)一级质谱同位素分布图、(c)二级质谱图和数据库中的甲氧苄氨嘧啶二级质谱图

为了考察本研究所建立的筛查方法的可靠性,采用已建立的一级精确质量数据库和二级谱图库对添加了86种化合物的渔药样品进行自动检索。86种化合物均与一级精确质量数据库检索匹配良好,质量偏差均小于5×10^-6^,检索得分均高于80。

### 2.5 方法学验证

#### 2.5.1 筛查限

本实验通过逐级稀释标准溶液浓度,将能够稳定可靠地筛查出化合物的最低浓度(筛查分数>80分)作为该化合物的筛查限^[20]^。中草药制剂取空白样品按1.4节将样品处理成空白基质溶液,使用空白基质溶液对标准溶液进行逐级浓度稀释;抗生素粉剂使用10%乙腈水溶液对标准溶液进行逐级浓度稀释。结果表明,中草药制剂和抗生素粉剂中86种禁限用药物非法添加的筛查限范围分别为1~15 mg/kg和5~50 mg/kg(具体数据见附表 https://www.chrom-China.com)。据文献^[5,6]^报道,渔药中禁限用药物添加浓度较高,本方法的筛查限可以满足实际检测需求。

#### 2.5.2 线性范围和定量限

根据86种化合物在质谱中的响应,配制不同浓度的系列混合标准溶液,中草药制剂采用空白基质溶液配制标准溶液,抗生素粉剂采用溶剂配制标准溶液。在1.5节条件下,将系列混合标准溶液上机测定。86种化合物均以提取离子色谱图的峰面积(*y*)为纵坐标,对应的质量浓度(*x*, μg/L)为横坐标绘制标准曲线,得到线性回归方程(具体数据见附表)。结果表明,在上述两种情况下,86种化合物的标准曲线均线性关系良好,相关系数(*r*^2^)均大于0. 99。

以目标化合物的信噪比等于10(*S/N*=10)的含量,确定该化合物的定量限(LOQ)(见附表)。结果表明,中草药制剂中86种化合物的定量限范围为1~15 mg/kg,抗生素粉剂中86种化合物的定量限范围为5~75 mg/kg。当渔药中禁限用药物添加含量较高导致初次上机浓度超出方法标准曲线的线性范围时,在1.4节所述的初始稀释倍数基础上,进一步稀释10、100、1000倍,以此类推,以10倍为数量级逐级稀释,直到待测物的上机浓度落在标准曲线的线性范围之内。其中,中草药制剂采用空白基质液稀释,抗生素粉剂采用10%乙腈水溶液稀释。通过稀释可满足实际过程中不同浓度禁限用药物添加的渔药筛查。本方法也适用于免疫调节剂、微生物制剂等其他多种渔药基质中禁限用药物的非法添加筛查,不同渔药基质筛查限略有不同。

### 2.6 回收率和精密度

采用在抗生素粉剂和中草药制剂基质中添加86种化合物标准溶液的方法进行添加回收率测定。中草药制剂中的添加浓度为20、100和150 mg/kg,抗生素粉剂中添加浓度为50、250和500 mg/kg,每个水平重复测定3次,按照1.4节前处理方法和1.5节仪器条件进行测定,筛查与定量结果表明,各药物的检出率为100%, 86种化合物在两种渔药中的加标回收率为76.8%~112.1%,相对标准偏差(RSD, *n*=3)均小于11.7%。由此可见,本实验建立的筛查方法准确可靠,可实现渔药中86种化合物的无标准品、高选择性的快速筛查和确证。

### 2.7 实际样品检测

应用本文所建立的筛查方法对浙江省主要水产养殖投入品质量安全抽检任务中的60批次渔药样品进行禁限用药物非法添加的快速筛查检测。在60个渔药样品中,1种标注主要成分为恩诺沙星的抗菌药中未检出有效成分,8种中草药中筛查出成分表中未标注的药物成分,具体结果见表2。由此可见,目前浙江省生产环节使用的渔药存在非法添加未注明药物、质量参差不齐等不良现象。因此,渔技推广部门需要加强对养殖户科学选药的指导宣传,渔政部门应加强渔药的监管工作,有效地制约不法市场行为,倡导科学用药,保证水产品质量安全。

**表2:** 实际筛查渔用中草药制剂阳性样品结果

Sample	Unlisted ingredient	Contents/(mg/kg)	Mass errors/10^-6^	Scores (TOF/MS)	Scores (TOF/MS/MS)
1	enrofloxacin	3515	-1.21	96	90
2	trimethoprim	1121	0.93	96	87
3	trimethoprim, enrofloxacin	452, 289	1.12, 0.84	92, 94	91, 89
4	ofloxacin, enrofloxacin	167, 243	0.77, 0.56	89, 91	84, 90
5	trimethoprim	2278	0.97	97	91
6	sulfamethoxazole	102	1.31	93	93
7	trimethoprim	512	0.56	95	86
8	enrofloxacin	23720	-0.54	95	91

## 3 结论

本研究利用液相色谱-四极杆-飞行时间质谱,建立了10类共86种化合物的精确质量数据库和二级质谱图库,针对不同渔药基质,通过优化稀释倍数进行前处理,配合数据库检索,实现了一次进样、不需要标准品即可完成渔药中86种禁限用药物非法添加的全面筛查与确证。方法准确、高效,可有效避免假阳性的出现,成功应用于浙江省渔药中禁限用药物非法添加的隐患排查。
